# Aberrant lipid metabolism in pulmonary inflammation linked to lung cancer progression; a preliminary study

**DOI:** 10.1186/s12967-023-04597-3

**Published:** 2024-06-06

**Authors:** Dongyoung Lee, Jinhee Mun, Injun Choi, Jinmyoung Joo

**Affiliations:** 1 Department of Biomedical Engineering, Ulsan National Institute of Science and Technology (UNIST), Ulsan, Republic of Korea; 2 Multi-Omics Research Center, PURIMEDI Corp., Seoul, Republic of Korea; 3 Graduate School of Health Science and Technology, Ulsan National Institute of Science and Technology (UNIST), Ulsan, Republic of Korea; 4 Center for Genomic Integrity, Institute for Basic Science, Ulsan, Republic of Korea

**To the Editor**,

Idiopathic pulmonary fibrosis (IPF), one of the main cause of lung cancer (LC), induces dysfunction of lipid metabolism (LM) into hypoxic condition [1]. In LCs, the biosynthesis of unsaturated fatty acids (UFAs) is dysregulated due to the hypoxia, whereas saturated fatty acids (SFAs) are increased to protect cells from oxidative stress [2, 3]. In particular, the dysregulation of UFAs can be quantitatively assessed with succinyl-CoA because it is the only coenzyme that can produce energy using UFAs under hypoxia [4]. Therefore, this study aims to compare lipid metabolite profiles according to the presence or absence of LCs, IPF through quantitative analysis of lipid metabolites.

We analyzed the difference in metabolite profiles among control, IPF, and LC groups through targeted lipid profiling in the sera of the mouse models, and the association between LM and cancer progression. In particular, short chain-alkanes (SCA, n = 8–20), a type of small lipid molecules produced from abnormal LM, were analyzed. A targeted analysis was conducted using headspace GC-MS in 10 controls, 11 IPFs, and 13 LCs in BALB/c and nude mice.

As a result, overall serum SCA levels were lower in the IPF than in control, but higher in LC. For SCAs above C15, there was no significant difference according to the presence of LC, and SCAs below C9 did not exceed the level of detection. However, the significance among three groups was shown in C10-14 (Fig. 1A). Interestingly, these targeted metabolites were consistently mentioned in a review discussing breath biopsy clinical trials [5].

**Fig. 1:**
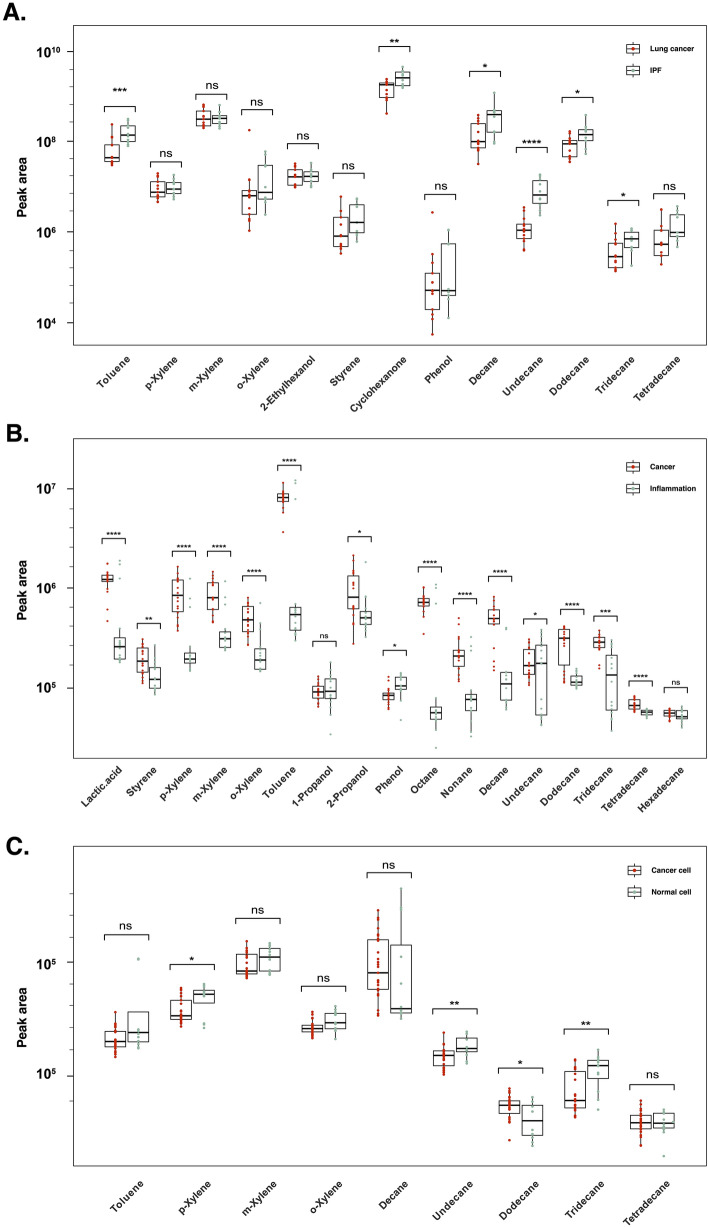
Targeted metabolite profiling using headspace gas chromatography and mass spectrometry (HS-GC‐MS). The box plot shows the log-scaled quantification of targeted metabolites.** A** Serum of mouse model: Lung cancer (n = 13; 6 nude mice, 7 balb/c mice), IPF (n = 11; 6 nude mice, 5 balb/c mice), and control (n=10; 5 nude mice, 5 balb/c mice).** B** Serum of human: Cancer (n = 20, rectal, colon, lung, stomach, liver, breast, ovarian, thyroid, and bile duct cancer) and inflammation (n = 16, encompassing hepatitis B and C, liver cirrhosis, Crohn’s disease, COPD, benign ovarian neoplasm, and Parkinsonism).** C** Cell media of cell culture: cancer (n = 27. CT26, Caki-1, MCF-7, OVCAR-3, MDA-MB-231, SKOV-3, DLD-1, ACHN, and A549) and normal (n = 9. CCD-18Co, Detroit551, and MRC-5). Note that the statistical significance (*p*-value) was obtained by t-test (ns:* p* > 0.05, *:* p* ≤ 0.05, **:* p* ≤ 0.01, ***:* p* ≤ 0.001, ****:* p* ≤ 0.0001)

This result indicates these LMs have significant association with cancer metabolites. Principal component analysis (PCA) for C10-14 SCAs showed advanced classification performance than when clustering all metabolites. However, the performance of odd chain SCAs (C11,13) showed complete performance, while that of even chain SCAs (C10, 12, 14) could not classify at all. This result is consistent with the fact that UFAs are the main energy source for succinyl-CoA in HLM. In addition, this is also consistent with the phenomenon in gene expression of LM among three groups in a previous study [2].

Next, we performed the same analysis in multiple cancer, noncancerous cells (nine, three cell lines, respectively), and clinical serum samples (8 inflammatory diseases, and 10 carcinoma comprising 80% of T1 to T2 stage and 20% of T3 stage in each cancer type) (Figs. 1B, C and 2). Interestingly, the results are also consistent with the in vivo experiment. Therefore, it seems that C10-14 SCAs are not lung cancer-specific biomarkers but characteristic metabolite biomarkers for multiple cancer detection. This study offers a novel technology for pan-cancer diagnostic approaches; however, it necessitates larger prospective studies to investigate the effect of inflammation on cancer development.

**Fig. 2:**
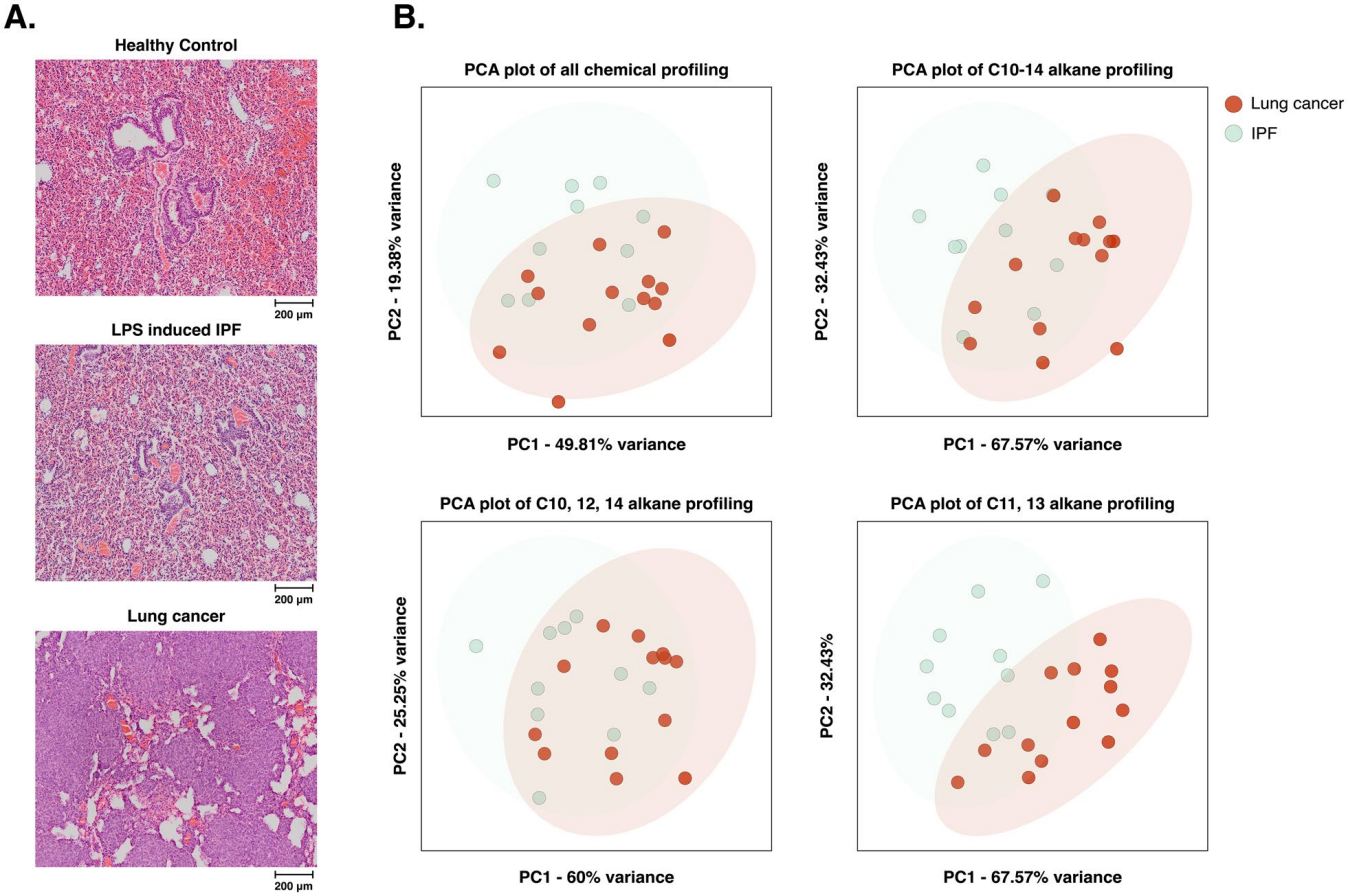
Unsupervised clustering analysis performance. **A** Representative H&E staining images depicting healthy control, LPS-induced IPF, and lung cancer groups in a mouse model. **B** PCA plots with 95% confidence ellipses showing targeted chemical profiling differences between lung cancer and IPF in all chemicals (untargted profiling including phenol, toluene, etc.), all SCA (C10-14), even (C10, 12, 14), and odd (C11, 13) SCA

## Data Availability

The raw data and detailed methodology can be provided upon request under authors’ consideration.
